# A Mechanistic Review of β-Carotene, Lutein, and Zeaxanthin in Eye Health and Disease

**DOI:** 10.3390/antiox9111046

**Published:** 2020-10-26

**Authors:** Fatima Tuj Johra, Asim Kumar Bepari, Anika Tabassum Bristy, Hasan Mahmud Reza

**Affiliations:** Department of Pharmaceutical Sciences, School of Health and Life Sciences, North South University, Bashundhara R/A, Dhaka 1229, Bangladesh; fatima.johra@northsouth.edu (F.T.J.); asim.bepari@northsouth.edu (A.K.B.); anika.bristy@northsouth.edu (A.T.B.)

**Keywords:** carotenoids, xanthophylls, eye disease, cataract, age-related macular degeneration, diabetic retinopathy, oxidative stress, zeaxanthin, lutein, β-carotene

## Abstract

Carotenoids are natural lipid-soluble antioxidants abundantly found as colorful pigments in fruits and vegetables. At least 600 carotenoids occur naturally, although about 20 of them, including β-carotene, α-carotene, lycopene, lutein, zeaxanthin, meso-zeaxanthin, and cryptoxanthin, are detectable in the human blood. They have distinct physiological and pathophysiological functions ranging from fetal development to adult homeostasis. β-carotene is a precursor of vitamin A that essentially functions in many biological processes including vision. The human macula lutea and eye lens are rich in lutein, zeaxanthin, and meso-zeaxanthin, collectively known as macular xanthophylls, which help maintain eye health and prevent ophthalmic diseases. Ocular carotenoids absorb light from the visible region (400–500 nm wavelength), enabling them to protect the retina and lens from potential photochemical damage induced by light exposure. These natural antioxidants also aid in quenching free radicals produced by complex physiological reactions and, consequently, protect the eye from oxidative stress, apoptosis, mitochondrial dysfunction, and inflammation. This review discusses the protective mechanisms of macular xanthophylls in preventing eye diseases such as cataract, age-related macular degeneration, and diabetic retinopathy. Moreover, some preclinical animal studies and some clinical trials are discussed briefly to understand carotenoid safety and efficacy.

## 1. Introduction

The major causes of progressive and irreversible loss of vision include various ophthalmic diseases such as cataract, age-related macular degeneration (AMD), glaucoma, and diabetic retinopathy. Initiation and progression of these disorders involve oxidative stress, apoptosis, mitochondrial dysfunction, and inflammation [1,2]. For instance, increased oxidative stress of retinal cells damages the mitochondrial DNA in diabetic retinopathy, one of the most deleterious eye-related complications of diabetes [3]. Oxidative stress is also a significant contributor to the pathophysiology of age-related cataract, a leading cause of blindness globally [2,4]. A growing body of evidence indicates that dietary antioxidants can prevent and treat many ophthalmic disorders associated with oxidative stress. Lutein, zeaxanthin, and meso-zeaxanthin (synthesized from lutein in the retina) are dipolar, terminally dihydroxylated carotenoids, also known as macular xanthophylls, and are obtained from dietary sources [5,6]. Macula lutea of the eye, also known as the yellow spot, contains high concentrations of macular xanthophylls. The peak concentrations of lutein and zeaxanthin appear at the center of the fovea [7]. Lutein and zeaxanthin are also found in the lens; however, β-carotene and lycopene have not been detected [8].

Carotenoids are the most abundant pigment groups and lipid-soluble antioxidants in nature that are responsible for the yellow, orange, or red color of fruits, leaves, and flowers [9,10]. They are C40-based isoprenoids of the tetraterpene family and are biosynthesized by the linkage of two C20 geranylgeranyl diphosphate molecules [11,12]. Stahl and Sies (2005) divided carotenoids into two classes: pro-vitamin A (e.g., β-carotene, α-carotene, and β-cryptoxanthin) and non-pro-vitamin A compounds [13]. Later, Jomova and Valko (2013) classified 600 naturally occurring carotenoids into three groups: carotenes, xanthophylls, and lycopene [14]. β-carotene, α-carotene, lycopene, lutein, and cryptoxanthin are some dietary carotenoids found in human blood [15]. β-carotenes, which are precursors of vitamin A, have greater pro-vitamin A potential compared to α-carotene or β-cryptoxanthin because of the presence of a β-ionone ring linked to a chain of 11 carbons [10,16]. β-carotene, α-carotene, and lycopene are composed only of carbon and hydrogen atoms, whereas xanthophylls are carotenoids with at least one oxygen atom. Zeaxanthin, lutein, α-and β-cryptoxanthin, canthaxanthin, and astaxanthin are important xanthophylls containing hydroxy and keto groups in their structures [13]. The presence of conjugated double bonds in the structure enables carotenoids to accept electrons from reactive species, neutralize free radicals, and isomerize and help in oxidation in the presence of oxygen, light, and heat [17]. Chemical reactivity, distinctive shape, and light-absorbing properties of carotenoids are attributed to the alternating double and single bonds present in the nucleus of a polyene chain that constitute a conjugated system with delocalized π-electrons. Different structural configurations and shapes exist because of the isomerism around C=C double bonds (e.g., *trans* or *cis* isomer) and possible rotation around C–C single bonds in the polyene chain. Compared to the *trans* isomers, *cis* isomers exhibit a higher structural instability owing to the steric hindrance between either hydrogen or methyl groups. Free rotation between the C-6 and C-7 single bonds in carotenoids allows them to twist and form an infinite number of possible angles between the ring structure and the main polyene chain. Carotenoids absorb light from the visible region in the wavelength range of 400–500 nm, promoting one of the π-electrons of the conjugated double to the previously unoccupied π* antibonding orbital [18]. Epidemiological studies identified the association of high dietary carotenoid intake with reduced risks of breast, cervical, ovarian, colorectal, cardiovascular, and eye diseases [19]. The U.S. Department of Agriculture reported that the average daily intake of lutein by Americans is about 1.7 mg per day, and in Europe, it is 2.3 mg per day. However, these values are far below the recommended dietary intake level of 6 to 14 mg per day to reduce the risk of macular degeneration and cataract [20].

## 2. Age-Related Macular Degeneration (AMD)

The amount of macular pigment is inversely associated with the incidence of AMD. Macular pigment levels can be improved by increasing the intake of foods rich in lutein and zeaxanthin (e.g., dark-green leafy vegetables) and supplementation. Lutein and zeaxanthin have been proved to be protective against the development of AMD in many studies [21,22].

**Figure 1:**
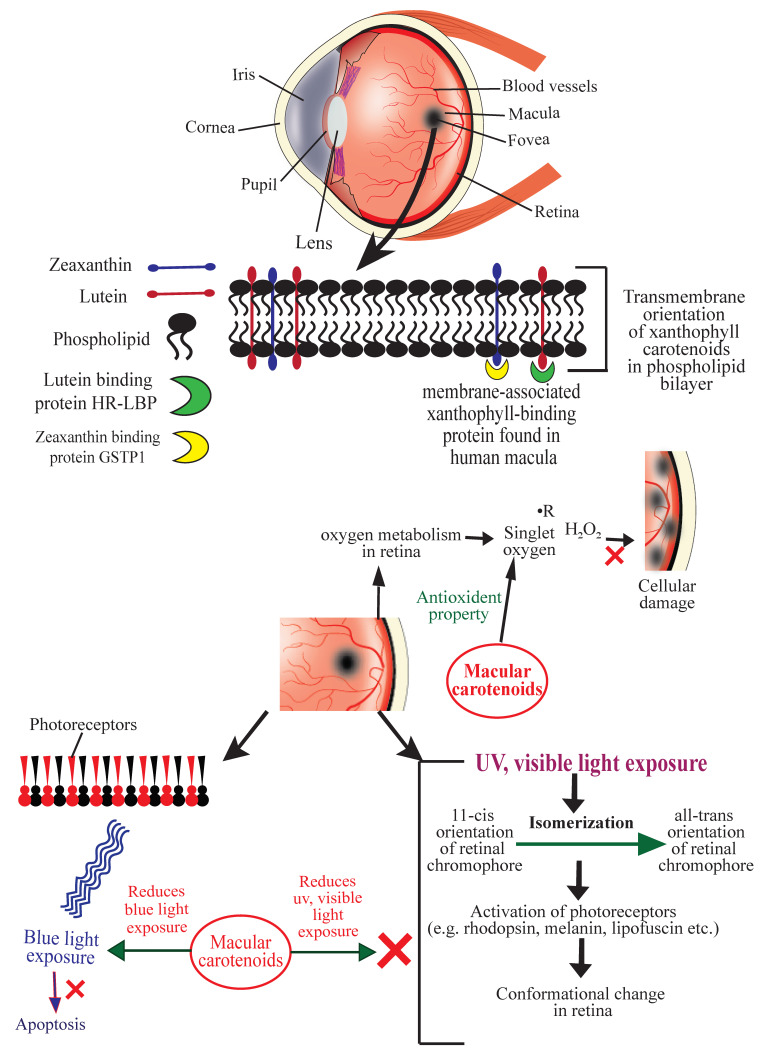
Schematic diagram showing the mechanisms of action of carotenoids to prevent age-related macular degeneration (AMD). HR-LBP: human retinal lutein-binding protein; GSTP1: glutathione S-transferase Pi 1; R: free radical (symbolic representation).

The retina of the human eye is abundantly supplied with oxygen. Prolonged or repeated exposure of light decreases long-chain polyunsaturated fatty acids in the retina, increases lipid conjugated dienes, and causes selective degeneration of photoreceptors and retinal damage [23]. Exposure of blue light in the retina is known to cause oxidative stress, mitochondrial and inflammatory apoptosis, and DNA damage; all of these lead to the development of glaucoma, keratitis, H and dry eye disease. [24]. Exposure of UV and visible light causes simultaneous photochemical isomerization of retinal chromophores and activation of photoreceptors (e.g., rhodopsin, melanin, lipofuscin, etc.) coupled with the chromophores. These events produce an electronic transition to the excited state and change chromophore structures from the 11-*cis* to the all-*trans* forms [25], and the retina undergoes a significant conformational change upon light absorption [26]. Reactive oxygen species (ROS) such as, free radicals, hydrogen peroxide, and singlet oxygen, generated by oxidative stress, cause cellular damage, promote the aging process in retina, and eventually lead to progression of AMD. For quenching these ROS and protecting retina from AMD, a particular transmembrane orientation of macular xanthophylls has been proposed [5]. Macular xanthophylls located transversely in the lipid bi-layer of the retinal membrane are able to prevent AMD and protect the retina against peroxidation and photo-damage by acting as antioxidants that quench free radicals and ROS [5]. They also prevent blue light exposure to fovea’s photoreceptors significantly [25] (Figure 1).

Bhosale et al. (2004) isolated and purified a membrane-associated xanthophyll-binding protein from human macula using ion-exchange chromatography and gel-exclusion chromatography. This protein is a Pi isoform of human glutathione *S*-transferase (GSTP1) to which zeaxanthin displayed the highest affinity. Uptake, metabolism, and stabilization of zeaxanthin in the retina were found to be mediated by this xanthophyll-binding protein [27]. The HR-LBP, a membrane-associated human retinal lutein-binding protein, displayed a saturable and specific binding toward lutein [28]. Once incorporated in the lipid bilayer, macular xanthophylls help quench singlet oxygen and other free radicals and thus prevent lipid peroxidation in the retina [29,30,31,32,33]. Carotenoids also protect against oxidative damage by repairing α-tocopherol and acting synergistically with vitamin C [23]. Figure 1 illustrates the mechanisms of action of ocular carotenoids to prevent AMD.

## 3. Cataracts

A cataract is a visualization problem in which the lens develops opacity, and age-related cataract is a leading cause of blindness. Depending on the morphology, cataract is classified into different types. The outer section of the tissue becomes opaque in cortical cataracts, the inner core in nuclear cataracts, and the superficial region below the capsule on the posterior side in posterior subcapsular cataracts [34]. In western countries, cataract surgery is most frequently done in people aged 65 years or older [35]. This is one of the most common surgical procedures among the general population, and the prevalence is increasing each year [36]. In the United States, 3.38 million cataract surgeries were performed in 2017 [37]. Although cataract is mainly an age-related phenomenon, socioeconomic and lifestyle factors (smoking, diet, intake of nutrients, alcohol consumption, etc.) also influence cataract initiation and progression [35,38].

**Figure 2:**
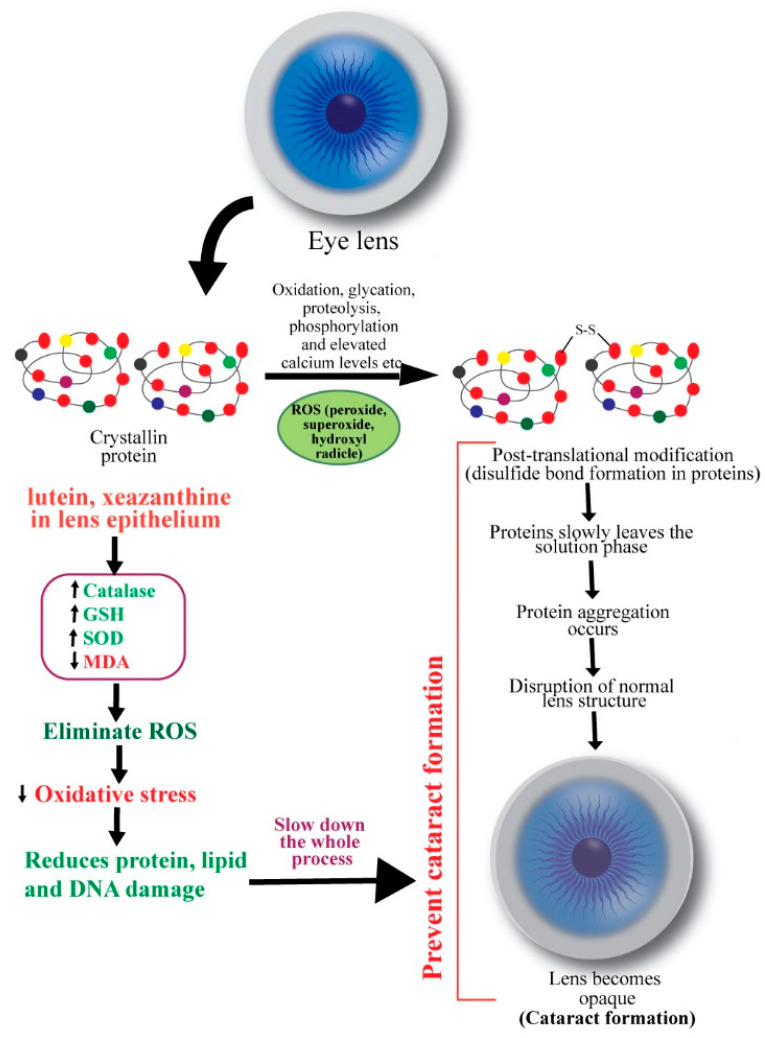
Schematic diagram showing the mechanisms of action of carotenoids to prevent cataract. ROS: reactive oxygen species.

The main constituents of an eye lens are crystallins (90%), and cytoskeletal and membrane proteins. Crystallins have a high refractive index and form a complex protein solution in the cytoplasm of lens fibers, conferring transparency. With age, this protein slowly leaves the soluble phase. Subsequently, disulfide bond formation and non-enzymatic glycation alter attractive forces between lens proteins [34] (Figure 2). Masters et al. (1977) observed aspartic acid racemization during aging and cataract formation on a D/L enantiomeric analysis of control human lenses and cataracts [39]. The insoluble fraction of D-aspartic acid becomes less abundant in cataractous lenses [40]. Thus, crystallins may undergo various post-translational modifications such as oxidation, glycation, proteolysis, transamidation, carbamylation, and phosphorylation [41]. These changes result in aggregation of proteins, disruption of healthy lens cell structure, and opacification.

Ocular oxidative stress may result from an imbalance between the generation of reactive oxygen species (ROS) and the cellular antioxidant defense mechanisms and subsequently initiate lens opacification [42]. ROS, such as hydrogen peroxide, superoxide, and hydroxyl radicals, negatively modifies the lens, whereas antioxidants, including glutathione (GSH), ascorbate, and catalase, rescue the lens proteins against ROS [43,44]. Hydrogen peroxide, the primary oxidant in the pathogenesis of cataract, is eliminated by catalase and glutathione through enzymatic reactions. A decreased level of reduced glutathione in older lenses’ nucleus promotes cataract formation [43,45]. An imbalance in redox reactions can also initiate lipid peroxidation, promoting cataractogenesis. Spector (1995) mentioned that the massive oxidation of thiol to protein and mixed disulfides, cysteic acid, and methionine sulfoxide and cataract-extensive methionine sulfoxide formation are common in older lens [34]. In the nucleus of nuclear cataracts, covalently linked disulfide bonds containing polypeptides and in cortical cataracts, high molecular weight disulfide-linked aggregates were found [46]. Thus, oxidation of crucial sulfhydryl groups of enzymes and membrane proteins and the peroxidation of lenticular plasma membrane lipids also contribute to cataract pathogenesis [47].

Carotenoids’ roles as antioxidants are known for many decades. β-carotene was found to markedly inhibit lipid peroxidation induced by xanthine oxidase in a pioneering study by Kellogg III and Fridovich [48]. Chemical antioxidants (e.g., α-tocopherol, β-carotene, ascorbate, and GSH) and structural antioxidants (e.g., cholesterol and membrane protein) are implicated in preventing oxidative damage of the ocular tissues [49]. Christen (1994) reviewed antioxidants’ protective effects in cataract and macular degeneration and found that animal studies invariably advocated in favor of dietary antioxidants, although results from epidemiological analyses were inconclusive [50]. The mechanisms of preventive functions of carotenoids in cataract formation are shown in Figure 2.

Human lens contains lutein and zeaxanthin but not β-carotene [51]. It has been suggested that antioxidants lutein and zeaxanthin are delivered continuously from the body pool to the epithelial/cortical layer of the lens, where they scavenge ROS by up-regulating GSH, catalase and SOD activities [52]. Gao et al. (2011) reported that lutein and zeaxanthin could reduce the risk for senile cataract by protecting lens protein, lipid, and DNA from oxidative damage. They incubated human lens epithelial cells with or without 5 µM lutein, zeaxanthin, or α-tocopherol for 48 h. Then the cells were exposed to 100 µM H_2_O_2_ for 1 h to induce oxidative stress. By using a battery of in vitro analyses, the authors observed that the levels of H_2_O_2_-induced protein carbonyl, MDA, and DNA damage were significantly reduced by lutein and zeaxanthin [53]. Interestingly, cataract patients exhibited increased serum levels of pro-oxidants and decreased levels of antioxidants. Serum level of MDA was significantly higher, and levels of superoxide dismutase (SOD) and glutathione peroxidase (GPX) were substantially lower in age-related cataract patients compared to healthy volunteers [54,55].

## 4. Diabetic Retinopathy

Glycemic control, diabetes duration, hypertension, hyperlipidemia, smoking, age, and genetic factors are responsible for developing microvascular complications like diabetic retinopathy, diabetic nephropathy, and diabetic neuropathy [56]. Diabetic retinopathy is prevalent in people with Type 1 and Type 2 diabetes mellitus. Glycated hemoglobin (HbA1c), a measure of mean glycemia, has been identified as a risk factor for the progression of diabetic retinopathy [57,58]. Carotenoids enhance insulin sensitivity and have a protective effect against diabetes-related infectious diseases [59].

**Figure 3:**
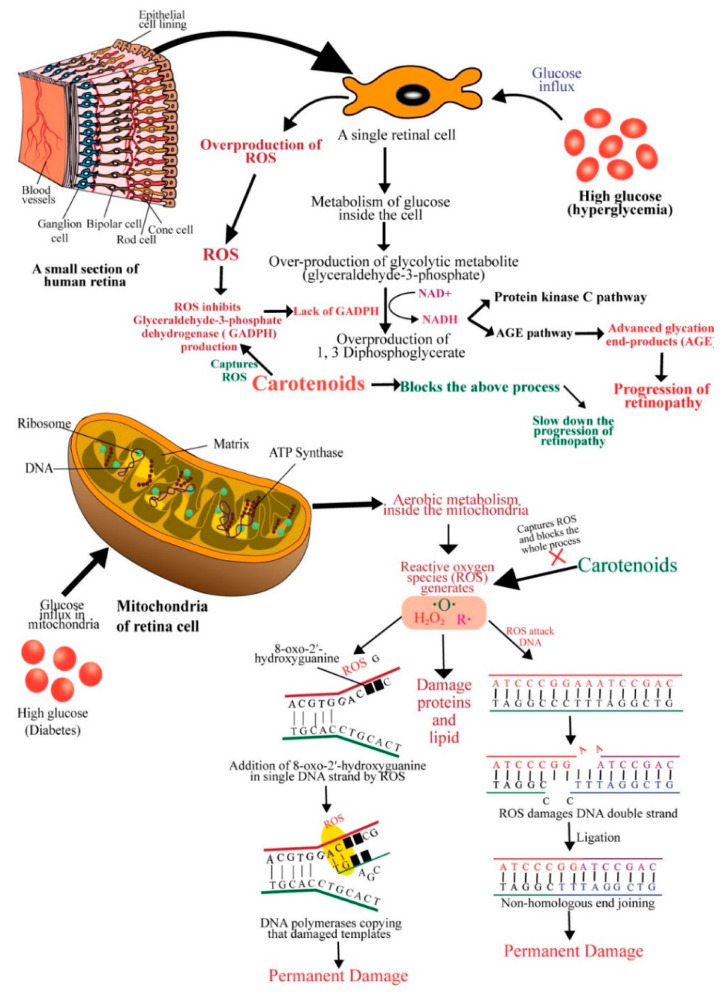
Schematic diagram showing the mechanisms of action of carotenoids to prevent diabetic retinopathy. ROS: reactive oxygen species; GADPH: glyceraldehyde-3-phosphate dehydrogenase; AGE: advanced glycation end-product; DNA: deoxyribonucleic acid; ATP: adenosine triphosphate.

In diabetes, the high glucose level present in the microvasculature of the retina compromises the electron transport chain system, produces superoxides, damages mitochondrial DNA and decreases proteins encoded by its DNA, and thus, causes metabolic, structural, and functional changes in the retina [60]. Hyperglycemia can initiate many biochemical changes in the retinal microvasculature, including increased oxidative stress in the polyol pathway, protein kinase C (PKC) activation, and advanced glycation end-product formation [61] (Figure 3). Rat retinal endothelial cells exposed to high glucose (HG) showed a down-regulation of the protein kinase B (also known as AKT) pathway and increased apoptosis [62]. HG was also found to increase mitochondrial fragmentation and pro-apoptotic cytochrome c levels in vascular cells of rat retinal capillaries [63,64]. Increased oxidative stress, elevated oxidatively modified DNA, and up-regulated nitrosylated proteins ensue an impairment in antioxidant defense enzymes, which eventually leads to increased retinal capillary cell apoptosis [65]. Further, mitochondrial metabolism generates ROS, such as superoxides and hydrogen peroxide, that can damage proteins, lipids, and DNA. The damage of proteins can be compensated because of continuous biosynthesis; however, DNA damage can be devastating if a fixed mutation occurs. If reactive oxygen species damage a portion of a single DNA strand (e.g., the addition of 8-oxo-2′-hydroxyguanine in DNA strand) and DNA polymerases copy that damaged templates during replication, then, this error becomes permanent [66,67,68] (Figure 3).

DNA double-strand can also be affected and broken by free radicals. This breakdown is usually repaired by ligating nonhomologous DNA ends, an error-prone repair system [68]. In an in vitro study, Santos, Tewari and Kowluru, (2012) observed that the damage caused by ROS was compensated by increased mitochondrial DNA biosynthesis and repair system in the early stages of diabetes (15 days to 2 months). At a stable diabetic condition (at 6 months of diabetes) with constant production of high ROS, mitochondrial DNA and electron transport chain (ETC) were damaged because repair/replication machinery became subnormal and mitochondrial DNA copy number was significantly decreased. An increase in apoptosis was also observed in the above study [69]. Compromised DNA repair machinery, decreased gene expressions of mitochondrial-encoded proteins, and increased mtDNA damage were observed at high glucose exposure of retinal endothelial cells [70]. Aso et al. (2000) observed a higher amount of advanced glycation end-products (non-enzymatic binding of glucose to free amino groups of an amino acid) in patients with retinopathy [71,72]. In hyperglycemic conditions, a high glucose level causes overproduction of a glycolytic metabolite glyceraldehyde-3-phosphate. Glyceraldehyde-3-phosphate can easily be converted into 1, 3 Diphosphoglycerate by converting nicotinamide adenine dinucleotide (NAD+) into to its reduced form (NADH) when ROS inhibits the overproduction of glyceraldehyde-3-phosphate dehydrogenase (GADPH). NADH facilitates the protein kinase C (PKC) pathway and the AGE pathway [73]. Advanced glycation end-products (AGEs) (glucosepane and methylglyoxal hydroimidazolone) were significantly associated with the progression of retinopathy [74].

Antioxidants such as ascorbate, tocopherol, and carotenoids protect ocular oxidative damage [75]. Carotenoids can quench free radicals, scavenge reactive oxygen species, modulate gene expression, reduce inflammation, and prevent diabetes-related microvascular complications, including diabetic retinopathy, nephropathy, and neuropathy [76]. Macular pigment (MP), including lutein, zeaxanthin and mesozeaxanthin also contributes to the protection of the retinal tissue by conferring potent antioxidant and anti-inflammatory effects in diabetes. It has been demonstrated that patients with type 2 diabetes have a lower level of MP as compared to healthy controls [77]. Lutein supplementation is known to prevent oxidative damage in the retina [78]. In a mouse model of early diabetic retinopathy, long-term lutein administration attenuated inflammation, and vascular damage of the retina [79]. Intriguingly, short-term lutein treatment also down-regulated reactive oxygen species and up-regulated superoxide dismutase (SOD), attenuating inflammation and protecting the photo-stressed retina from oxidative damage [80]. Several signaling pathways, including PKC, vascular endothelial growth factor (VEGF), nuclear factor erythroid 2-related factor 2 (Nrf2), and Rho/Rho-associated coiled-coil containing protein kinase (Rho/ROCK), have been implicated in carotenoid-mediated protection of the retina in diabetic retinopathy [81]. An in vitro study showed that co-administration of lutein and zeaxanthin attenuated VEGF-induced oxidative stress in the retinal endothelium [82]. Lutein was found to modulate the SIRT1 signaling and inhibit premature senescence in retinal pigment epithelium cells [83]. Recent studies indicate that carotenoids could exert therapeutic benefits in diabetic retinopathy through multiple cellular and molecular pathways. The mechanisms of action of carotenoids to prevent diabetic retinopathy discussed above are sketched in Figure 3.

## 5. Safety of Carotenoids

Several lines of evidence have demonstrated the safety profiles of carotenoids supplementation at different doses and duration in experimental animals. Table 1 summarizes some of these outcomes.

## 6. Clinical Trials

Clinical trials are the final step assessments of any drug before it is approved for regular human application. Twenty-six (26) important clinical trials are summarized in Table 2 to understand the efficacy of carotenoids as prophylactic and therapeutic uses in eye diseases.

Preclinical study results listed in Table 1 have established the safety profile of several carotenoids. Results from different clinical trials listed in Table 2 confirm that an increased serum level of lutein was correlated with enhanced visual acuity [93,96,116]. An increase in macular pigment optical density (MPOD) was seen with lutein and zeaxanthin supplementation [95,96,97,98,99,100]. Several studies reported that lutein and zeaxanthin administration was associated with a reduced risk of cataract [101,102,103,104,105,114]. Nonetheless, some studies did not find statistically significant effects of lutein and zeaxanthin on prevention of eye diseases or enhancement of macular pigments [99,102,113,115]. In a study by Manayi et al. (2015), lutein and zeaxanthin were found in the lens, but β-carotene and lycopene were not detected [8]. Among six clinical trials mentioned in Table 2, which examined the effects of β-carotene on cataract, five found no significant impact [104,107,109,112], and one showed a small reduction in the progression of age-related cataract [111]. A study found that smokers, people with high BMI, a history of hypertension, diabetes, and high cholesterol developed cataracts even after taking antioxidants [101]. These studies suggest that β-carotene is not adequate for cataract prevention as the lens does not contain any β-carotene. On the contrary, in vivo studies on animals showed that lutein is safe even at a very high dose, and the LD_50_ of lutein exceeded 10,000 mg/kg body weight [86]. Eight in vivo studies were mentioned in this review (Table 1), and none of these studies observed any significant adverse effect or toxicity. Xu et al. (2013) suggested a daily intake of 3 mg/kg/day meso-zeaxanthin for human [88]. Intake of up to 20 mg/day for lutein was found to be safe for humans [117].

## 7. Conclusions

In this review, we summarized the detrimental effects of ocular oxidative stress generated from the continuous exposure of ultraviolet and blue lights. Then we discussed the protective roles of carotenoids, namely β-carotene, lutein, and zeaxanthin, against three distinct eye diseases, highlighting the outcomes from the clinical trials. A considerable number of studies including preclinical and clinical trials demonstrated that β-carotene, lutein, and zeaxanthin can prevent the progression of eye diseases, mainly by quenching free radicals and preventing oxidative damage to the retina. According to study outcomes, it is obvious that β-carotene, lutein, and zeaxanthin can efficiently attenuate oxidative stress in vivo and confer protection to the eye.

The biological functions of different carotenoids in human are established. As the human body cannot synthesize this important class of molecules, they must be supplied as dietary intake or food/pharmaceutical supplement. Thus, the optimum levels of cellular concentrations of β-carotene, lutein, and zeaxanthin in eye tissue may help maintain eye health. It is noteworthy that carotenoids, particularly MP, can be assessed noninvasively in retina; such assessment may be useful to determine the average dietary intake of lutein and zeaxanthin to meet the regular need of these molecules [118]. It has been shown that MP attenuates oxidative stress and slows down the progression of apoptosis, mitochondrial dysfunction, and inflammation in diabetes, which can be improved by increasing dietary supplementation of lutein and zeaxanthin [77].

A long-term cohort study by Wu et al. (2015) found a remarkable 40% reduced risk of advanced AMD progression for predicted plasma lutein/zeaxanthin scores [119]. Nevertheless, carotenoids show a high degree of variability in bioavailability, which poses a challenge for finding suitable forms (as foods, supplements, or medicines) that can be administered to the patients with AMD. Gastrointestinal absorption and subsequent distribution to ocular tissues are influenced by dietary factors, formulations, gender, age, disease states, and individual genetic variations [120,121]. A recent study found a significantly higher absorption of zeaxanthin and meso-zeaxanthin from a diacetate micromicelle preparation than free carotenoid preparations [120]. Intriguingly, a nano-formulation of lutein-poly-(lactic-co-glycolic acid) (PLGA)-phospholipid (PL) showed a significantly elevated level of lutein in plasma when administered at a lower dose in mice [87]. Novel drug delivery systems and formulations thus could further be exploited to achieve favorable pharmacokinetic and pharmacodynamic profiles of macular xanthophylls in humans. In addition, long-term clinical trials with large numbers of populations may be undertaken to confirm the effects of these molecules. Future studies will substantiate the therapeutic potentials of different β-carotenoids.

## Figures and Tables

**Table 1:** In vivo preclinical studies to assess the safety of carotenoids.

Reference	Type and Number of Animal Used	Duration of Therapy	Name and Dose of Carotenoids Administered	Parameters Observed	Findings and Observation
[84]	120 wistar strain rats (60 males and 60 females)	90 days	Lutein/zeaxanthin at the concentrate of 0, 4, 40, and 400 mg/kg body weight/day. It was given as extract of marigold flowers (*Tagetes erecta* L) containing minimum 80% carotenoids of which 67% is lutein and 13.5% is zeaxanthin isomers.	Body weights, urine parameters (volume, specific gravity, color, clarity, pH, RBC, WBC, bilirubin, ketone bodies, proteins, glucose and nitrite), hematological analysis, serum parameter analysis (glucose, urea, creatinine, cholesterol, triglycerides, AST, ALT, ALP, bilirubin, sodium, potassium, chloride, total protein, albumin, globulin and A/G ratio), and gene mutation	No adverse effect was observed. NOAEL: 400 mg/kg/day HED: 64.8 mg/kg/day
[85]	20 rats (10 male, 10 female)	90 days	Lutein diacetate 2.1, 22.5, and 210 mg/kg body weight/day	Body weights, net body weight gains, feed intake, neurological observations, hematology and clinical chemistry	No adverse effect was observed. It is relatively safe in rats up to this dose. NOAEL: 210 mg/kg/day HED: 34.02 mg/kg/day
[20]	70 wistar rats (35 males and 35 females) for short-term toxicity study and 70 more rats for subchronic toxicity study	Short-term toxicity study: 4 weeks, subchronic toxicity study: 13 weeks	Lutein and its ester was administered orally at doses of 4, 40, and 400 mg/kg body weight/day	Body weight, food consumption pattern, organ weight, other adverse side reactions, alteration in hepatic and renal function, change in the hematological parameters and in lipid profile	No adverse effect was observed. No mortality was produced by lutein and lutein ester up to a concentration of 4 g/kg. Lutein and its ester form was found nontoxic. NOAEL: 400 mg/kg/day HED: 64.8 mg/kg/day
[86]	80 weanling male albino mice (50 for acute toxicity study, 30 for subacute toxicity study)	Acute toxicity study: 14 days, subacute toxicity study: 4 weeks	Lutein 0.57, 100, 1000, and 10,000 mg/kg body weight for acute toxicity study; 0, 100, and 1000 mg/kg body weight/day for subacute toxicity study	Clinical observation, ophthalmic examinations, body, organ weights, hematological, histopathological, and other clinical chemistry parameters	LD50 exceeded 10,000 mg/kg body weight. No toxicity was observed up to this dose. NOAEL: 1000 mg/kg/day HED: 81 mg/kg/day
[87]	90 female swiss albino mice (50 for acute toxicity study, 40 for subacute toxicity study)	Acute toxicity study: 2 weeks, subacute toxicity study: 4 weeks	Lutein-poly-(lactic-co-glycolic acid) (PLGA)-phospholipid (PL) nano-capsules 0.1, 1, 10, and 100 mg/kg body weight for acute toxicity study; 1 and 10 mg/kg body weight/day for subacute toxicity study	Mortality, ophthalmic examinations, body and organ weights, hematology, histopathology and other blood and tissue clinical chemistry parameters	No toxicity or adverse effect was observed at a dose of 10 mg/kg body weight. NOAEL: 10 mg/kg/day HED: 0.81 mg/kg/day
[88]	100 SD rats	Subchronic toxicity: 90 days, acute toxicity: 2 weeks	Maximum tolerable dose was more than 10.0 g/kg body weight (acute oral toxicity tests), Highest dosage of 300 mg/kg/day meso-zeaxanthin	Acute toxicity, genetic toxicity (Ames test, mice bone marrow erythrocyte micronucleus and mice sperm abnormality)	No acute toxicity and no genotoxicity was observed. NOAEL: 300 mg/kg/day HED: 48.6 mg/kg/day
[89]	120 wistar rats (60 males and 60 females)	90 days	Zeaxanthin concentrate at doses of 0, 4, 40, and 400 mg/kg body weight/day for sub-chronic toxicity study; additional 100, 200, 400, and 1000 mg/kg body weight/day; 4 rats were given zeaxanthin 2000 mg/kg bw/day for acute toxicity study	Body weight, feed consumption; ophthalmoscopic examination, clinical pathology, neurological examination, clinical chemistry, hematology, urine analysis, necropsy, organ weight, histopathology, mutagenic activity	No mortality, toxicity, and mutagenicity was observed. NOAEL: 400 mg/kg/day HED: 64.8 mg/kg/day
[90]	50 han wistar rats	13 weeks followed by a 4-week recovery period	Meso-zeaxanthin 2, 20, and 200 mg/kg/day.	Potential toxicity and genotoxicity	No adverse effect was observed up to 200 mg/kg/day. NOAEL: 200 mg/kg/day HED: 32.4 mg/kg/day

NOAEL: no observed adverse effect level; HED: human equivalent dose (calculated according to the USFDA guideline); RBC: red blood cell; WBC: white blood cell; AST: aspartate aminotransferase; ALT: alanine aminotransferase; ALP: alkaline phosphatase.

**Table 2:** Clinical trials of carotenoids against eye disease.

Reference	Trial ID	Study Type	Study Duration	Characteristics of Participants	Intervention Group(s)/Comparison Group(s)/Assessment Criteria	Result
[91]	NCT00345176	Multicenter, randomized, double-masked, placebo-controlled phase 3 study	7 years	Patients N = 4203 Age range: 50–85 years Age (mean ± SD): 73.1 ± 7.7 Sex: 56.8% Female	Primary randomization: G 1: AREDS formulation G 2: AREDS, L 10 mg, Z 2 mg G 3: AREDS, DHA 350 mg, EPA 650 mg G 4: AREDS, L 10 mg, Z 2 mg, DHA 350 mg, EPA 650 mg	No statistically significant reduction in progression to advanced AMD was observed (*p* = 0.12 for L + Z).
					Secondary randomization: G 1: AREDS supplement G 2: AREDS supplement with no beta carotene G 3: AREDS with low dose zinc (25 mg) G 4: AREDS supplement with no beta carotene and with low-dose zinc	
[92]	ISRCTN60816411	Single-blind, randomized controlled clinical trial	3 years	Patients *N* = 67 (52 completed Age (mean ± SD): 66 ± 8 Sex: 65.4% Female	G 1: 20 mg L, 0.86 mg Z G 2: 10 mg MZ, 10 mg L, 2 mg Z G 3: 17 mg MZ, 3 mg L, 2 mg Z	No statistically significant change in AMD grade between intervention groups (*p* = 0.29, Fisher’s exact test).
[93]	ISRCTN 94557601	Randomized double-masked placebo-controlled clinical trial	Each participant was followed up for up to 3 years	Patients *N* = 433 Age range: 50–95 years Age (mean ± SD): 75.9 ± 7.7 Sex: 57.3% Female	G1: A tablet was taken twice daily to deliver a daily dose of 12 mg L, 0.6 mg Z, 15 mg d-α-tocopherol, 150 mg ascorbic acid, 20 mg zinc oxide, and 0.4 mg copper gluconate G2: Placebo (made up of cellulose, lactose, and magnesium stearate)	4.8 letters better BCVA was seen in active group than placebo group, which was statistically significant (*p* = 0.04). Visual acuity increased by 1.4 letters with 1-log-unit increase in serum lutein. Morphologic severity progressed slowly.
[94]	ISRCTN 17842302	Randomized clinical trial on participants with AMD	60 weeks	Patients *N* = 14 Age range: 56–83 years Age (mean ± SD): 67.3 ± 8.5	G1: Ascorbic acid 150 mg, cupric oxide 400 µg, DL-α-tocopherol 15 mg, zinc oxide 20 mg, lutein 12 mg, Z 0.6 mg, EPA 240 mg, DHA 840 mg G2: No supplement	A nonsignificant latency and reduced amplitudes of multifocal electroretinography (mfERG) was seen on supplement withdrawal (ring 3 N2 latency, *p* = 0.041 and ring 4 P1 latency, *p* = 0.016).
[95]	-	Double-blind and placebo-controlled clinical trial	140 days	Patients *N* = 100 Age range: 18–64 years Sex: 48% Female	G1: Placebo G2: 5 mg L G3: 10 mg L G4: 20 mg L (younger group) G5: 20 mg L (older group)	A linear, dose-dependent increase in macular pigment optical density (MPOD) was observed (*p* < 0.0001).
[96]	NCT00763659	A prospective, randomized double-blind, placebo controlled clinical trial on patients with non-exudative AMD	12 months	Patients *N* = 172 Age (mean ± SD): 70 ± 10 Sex: 54.7% Female	G1: 10 mg L, 1 mg Z, 255 mg concentrated fish oil (100 mg DHA, 30 mg EPA), 60 mg vitamin C, 20 mg vitamin E, 10 mg zinc, 0.25 mg copper G2: 20 mg L, 2 mg Z, 500 mg concentrated fish oil (200 mg DHA, 60 mg EPA) and 120 mg vitamin C, 40 mg vitamin E, 20 mg zinc, 0.5 mg copper G3: Placebo	Statistically significant increase in MPOD was seen in both G1 and G2 (*p* < 0.001). Supplementation with L and Z improved and stabilized BCVA in AMD patients.
[97]	-	Clinical trial on patients with unilateral wet AMD and patients with unilateral cCSC	6 months	Patients *N* = 20 Age: >56 years Age (mean ± SD): 66 ± 4 Sex: 30% Female	Sante Lutax 20 plus DHA (20 mg L, 1 mg Z, and 200 mg DHA)	MPOD (*p* = 0.032) and contrast sensitivity (*p* < 0.05) improved. L, Z, DHA dietary supplement had beneficial effects.
[98]	NCT00909090	Randomized, double-blind, placebo-controlled clinical trial	400 days	Patients *N* = 115 (109 completed) Age (mean ± SD): 23.2 ± 4 Sex: 59.6% Female	G1: 10 mg L, 2 mg Z G2: Placebo	MPOD (*p* < 0.0001), chromatic contrast (*p* < 0.0001) and photostress recovery time (*p* = 0.002) improved significantly.
[99]	NCT10528605	Randomized, double-blind, placebo controlled trial on patients with early AMD	2 years	Patients *N* = 112 Age: >50 years Age (mean ± SD): 69.1 ± 7.3 Sex: 57.4% Female	G1: Placebo G2: 10 mg L G3: 20 mg L G4: L 10 mg, Z 10 mg	MPOD, retinal sensitivity, N1P1 response densities in ring 1 and ring 2 increased significantly in G2, G3, G4 (all *p* < 0.05)
[100]	ISRCTN54990825	A double-blind, placebo-controlled	12 weeks	Healthy subjects *N* = 32 Age range: 18–25 years	G1: Placebo G2: 6.18 mg L, 0.73 mg Z, 0.53 mg MZ (total 7.44 mg) G3: 10.86 mg L, 1.33 mg Z, 0.94 mg MZ (total 13.13 mg) G4: 22.33 mg L, 2.70 mg Z, 2 mg MZ (total 27.03 mg)	Serum level increased linearly with increased dose. Group 3 showed the highest ratio of MPOD change, which was statistically significant (*p* = 0.021).
[101]	-	Randomized, double masked, placebo-controlled trial	10 years	Patients and healthy subjects *N* = 39,876 Age range: ≥45 years Age (mean): 53.5 (no cataract), 61 (cataract) Sex: 100% Female	β-carotene, vitamin A, and vitamin E	Only 2031 people developed cataract. Most of them were smokers, had high BMI, and reported a history of hypertension, diabetes, and high cholesterol. Higher dietary intakes of L/Z (*p* = 0.04) and vitamin E (*p* = 0.03) significantly decreased the risk of cataract.
[102]	-	Prospective study	5 years	Patients and healthy subjects *N* = 400 Age range: 50–86 years Sex: Both male and Female	No supplement was provided. Serum concentrations of individual carotenoids, α- and γ-tocopherol were determined	Serum carotenoids did not have significant association with nuclear cataract prevention (*p* = 0.13).
[103]	-	Cohort study	10 years	Patients and healthy subjects *N* = 5925 Age range: 43–84 years Sex: Both male and Female	Intake of L and Z was assessed using a food frequency questionnaire	L (intake in the distant past) had protective influence on the development of nuclear cataracts (*p* = 0.002).
[104]	-	Prospective study	8 years	Patients and healthy subjects *N* = 36,644 Age range: 45–75 years Sex: 0% Female	Dietary questionnaire was used to assess	A statistically significant lower risk of cataract in higher intakes of L and Z was observed (*p* = 0.03). No impact of other carotenoids (α-carotene, β-carotene, lycopene, and β-cryptoxanthin) are found in cataract prevention
[105]	-	Prospective cohort study	12 years	Patients and healthy subjects *N* = 77,466 Age range: 45–71 years	Food frequency questionnaire was used to assess	Foods rich in carotenoids (L and Z) decreased the risk of cataracts (22%) (*p* = 0.04).
[106]	NCT00000145	Clinic-based, baseline cross-sectional and prospective cohort study	9.6 years	Patients *N* = 3115 Age range: 55–80 years Sex: Both male and Female	Food frequency questionnaires	No significant association of L plus Z intake with the development of nuclear or cortical lens opacity.
[107]	NCT00000145	11-center age-related eye disease study double-masked clinical trial	6.3 years	Patients *N* = 4757 Age range: 55–80 years Sex: 56% Female	G1: Antioxidants (β-carotene 15 mg, vitamin C 500 mg; vitamin E, 400 IU) G2: Antioxidants (vitamin C, 500 mg; vitamin E, 400 IU; and β-carotene, 15 mg) + zinc 80 mg + copper 2 mg G3: Zinc 80 mg + copper 2 mg G4: Placebo	No statistically significant effect was observed on the progression or prevention of cataract, age-related macular degeneration, age-related lens opacities, or visual acuity loss.
[108]	NCT00345176	Randomized, double-masked, controlled clinical trial, cohort study.	5 years	Patients *N* = 4203 Age range: 50–80 years Sex: 56.8% Female	G1: Control group G2: L 10 mg and Z 2 mg G3: Docosahexaenoic acid (DHA 350 mg) and eicosapentaenoic acid (EPA 650 mg) G4: L 10 mg and Z 2 mg, docosahexaenoic acid (DHA 350 mg) and eicosapentaenoic acid (EPA 650 mg)	Carotenoid supplementation did not have any significant positive or negative impact on the high mortality rate observed in people having age-related macular disease.
[109]	-	Randomized, double-masked, placebo-controlled trial	12 years	Patients and healthy subjects *N* = 22,071 Age range: 40–84 years Sex: 0% Female	β-carotene (50 mg) or placebo	β-carotene supplementation had no overall beneficial or harmful effect on cataract or cataract extraction.
[110]	NCT00000479	Randomized, double masked, placebo-controlled trial	2.1 years	Patients and healthy subjects *N* = 39,876 Age: ≥45 years Sex: 100% Female	G1: β-carotene G2: Placebo	No large beneficial or harmful effect was found on the development of cataract.
[111]	-	Multi-centered, prospective, double-masked, randomized, placebo-controlled trial	3 years	Patients *N* = 445 Age (mean ± SD): 66.2 ± 8.9 Sex: 59.3% Female	G1: 6 mg β-carotene, 200 mg α-tocopherol acetate (Vitamin E) and 250 mg ascorbic acid G2: Placebo	A small, statistically significant reduction in the progression of age-related cataract was observed after three years of treatment (*p* = 0.048).
[112]	NCT00342992	Randomized, double-blind, placebo-controlled clinical trial	5 to 8 years (median 6.6 years)	Patients *N* = 1828 Age range: 50–69 years Sex: 0% Female	G1: α-tocopherol 50 mg/day, G2: β-carotene 20 mg/day, G2: A combination of the two G4: placebo	No influence of supplementation on cataract prevalence.
[113]	NCT0140845	Prospective, randomized, double-masked multicenter study	24 months	Patients *N* = 126 Age (mean ± SD): 75.3 ± 7.6 Sex: 58.7% Female	G1: Carotenoids (5 mg of L and 1 mg of Z) + (560 mg of DHA, 420 mg GLA, 80 mg of vitamin C, 10 mg of vitamin E, 2 mg of vitamin B6, 200 g of Vitamin B9, 1 g of vitamin B12, 10 mg of Zinc) G2: Placebo + (560 mg of DHA, 420 mg GLA, 80 mg of vitamin C, 10 mg of vitamin E, 2 mg of vitamin B6, 200 g of Vitamin B9, 1 g of vitamin B12, 10 mg of Zinc)	Carotenoid supplementation did not improve MPOD, which was exacerbated by cataract and age-related macular degeneration.
[114]	-	Randomized, double-blind, placebo-controlled supplementation study	2 years	Patients *N* = 17 Age range: 55–73 years Sex: 86.7% Female	G1: 12 mg of all-trans-lutein, 3 mg of 13/15-cis-lutein, 3.3 mg of alpha-tocopherol G2: 100 mg of alpha-tocopherol G3: Placebo (0.06 mg of alpha-tocopherol, 0.23 mg of gamma-tocopherol, 500 mg of corn oil)	L has beneficial effect on age-related cataracts, whereas alpha-tocopherol was not beneficial.
[115]	NCT01269697	Phase 3, double-blind, randomized clinical trial	6 months	Healthy subjects *N* = 120 Age range: 40–70 years Age (mean ± SD): 56.7 ± 6.6 Sex: 71.7% Female	G1: L 5 mg, Z 1 mg, vitamin C (90 mg), vitamin E (15 mg), zinc (7.5 mg), copper (<0.5 mg), and resveratrol (0.5 mg), 33 mg of fish oil (50% ω-3) G2: Placebo	An increase in plasma L and Z concentrations was observed (*p* < 0.005) but no elevation of MPOD was found.
[116]	NCT00029289	Double-masked randomized placebo-controlled clinical trial with a crossover design	24 weeks	Patients *N* = 34 Age (mean ± SD): 49.2 ± 9 Sex: 61.8% Female	G1: L 10 mg/d for 12 weeks, 30 mg/d for next 12 weeks, followed by placebo for 24 weeks G2: Placebo 24 weeks, followed by L 10 mg/d for 12 weeks, followed by 30 mg/d for next 12 weeks	Significantly improved visual field (*p* = 0.038), slightly improved visual acuity. L 10–30 mg/day for up to 6 months was safe.

AREDS: Age-Related Eye Disease Study; AREDS formulation: vitamin 500 mg, vitamin E 400 IU, β-carotene 15 mg, zinc 80 mg (as zinc oxide), and copper 2 mg (as cupric oxide); BCVA: best-corrected visual acuity; ISRCTN: International Standard Randomized Controlled Trial Number; mfERG: multifocal electroretinography; ARM: age-related maculopathy; AMD: age-related macular degeneration; EPA: eicosapentaenoic acid; DHA: docosahexaenoic acid; BCVA: best-corrected visual acuity; GLA: gamma linolenic acid; MPOD: macular pigment optical density; L: lutein; Z: zeaxanthin; MZ: mesozeaxanthin; ARM: age-related maculopathy; cCSC: chronic central serous chorioretinopathy; BMI: body mass index.
